# The effect of prescription patterns on the performance of the pharmacy department of a Regional Referral Hospital, Uganda

**DOI:** 10.1080/20523211.2024.2306852

**Published:** 2024-02-20

**Authors:** Gerald Manzi Mbabazize, Vedaste Kagisha, Kato J. Njunwa, Joseph Oloro

**Affiliations:** a College of Medicines and Health Sciences, Regional Center of Excellence for Vaccines, University of Rwanda, Kigali, Rwanda; b Department of Pharmacy, Mbarara Regional Referral Hospital, Mbarara, Uganda; c Department of Pharmacology and Therapeutics, Faculty of Medicine, Mbarara University of Science and Technology, Mbarara, Uganda

**Keywords:** Medicines, prescription practices, pharmacy performance, rational drug use

## Abstract

**Background:**

Poor drug prescription patterns (PP) result in irrational medicine use, avoidable stock outs and drug expiries.

**Objective:**

This study primarily assessed the effects of PP on the performance of the pharmacy department (PD) of Mbarara Regional Referral Hospital (MRRH) Uganda.

**Methods:**

This was a mixed method cross-sectional study conducted in the outpatient department (OPD) of MRRH, questionnaires were administered to 86 prescribers and 300 patient prescriptions were reviewed. Ethical clearance was granted and informed consent of patients. Data were analysed, presented in the form of graphs, tables.

**Results:**

The overall prescription fill rate was 60.5%, being higher among dental officers and lower among those who did not adhere to generic prescribing and EML. Medical officers made most prescriptions at 69.3%. Prescriptions with four (47.1%) and six (17.5%) medicines respectively were made by clinical officers. Of the 300 prescriptions, 76% adhered to the Essential Medicines List (EML), 62% used generic name including 87.3% from dental officers and 52.9% by clinical officersThe overall prescription fill rate was 60.5%, being higher among dental officers and lower among those who did not adhere to generic prescribing and EML. Medical officers made most prescriptions at 69.3%. Prescriptions with four (47.1%) and six (17.5%) medicines respectively were made by clinical officers. Of the 300 prescriptions, 76% adhered to the Essential Medicines List (EML), 62% used generic name including 87.3% from dental officers and 52.9% by clinical officers.

**Conclusion:**

Prescription pattern affected the performance of the PD of MRRH, calling for its continued monitoring to ensure that guidelines are upheld, EML and UCG are availed and utilized.

## Introduction

Appropriate use of medicines is essential for the attainment of universal health coverage, as it ensures quality, safety, equity and affordable cost of care, particularly in low- and middle-income countries (Al Hamid et al., 2014; Wagner et al., 2014). The pharmacy department in a hospital has a big role to play in providing the required medicines to the patients, but its performance is greatly influenced by the prescription pattern (PP) of medicines (Adisa et al., 2016). The PP is a universal challenge that cuts across high-, middle- and low-income countries (Shanmugapriya et al., 2018; Shukar et al., 2021); and it is most evidenced by the noticeable failures in the supply chain of antibiotics, anaesthetics and anti-cancers (WHO, 2015).

Monitoring studies on medicine PP using the WHO drug use indicators (WHO, 1993) have been conducted in several countries including Nepal, South Africa, Ethiopia and Zambia; and they mostly reported that health workers did not fully adhere to the regulatory agency guidelines (Anagaw et al., 2023; Barth et al., 2016; Govender & Suleman, 2021; Niaz et al., 2020; Shrestha & Prajapati, 2019; Vledder et al., 2019; Wiedenmayer et al., 2021). The World Health Organisation (WHO) reports indicate that more than half of all drugs are prescribed and issued inappropriately (Garg et al., 2014). Consequently, this leads to irrational use of drugs, stock-outs, expiries, inefficient use of financial resources, treatment failure and antimicrobial resistance (Action Programme on Essential Drugs and Vaccines, 2010; Alex, 2018; Chem et al., 2018; Getahun et al., 2020; Ofori-Asenso & Agyeman, 2016; World Health Organization, 2023); and patients and the community suffer economic burden prompted by the use of costly medicines (UBOS-Statistical Abstract, 2020).

In response to the medicines PP challenges experienced, the WHO provided guidelines that spell out the required prescription patterns (Cotter, 1995; Jain et al., 2015) which stipulate that prescriptions should be written in line with clinical guidelines, EML, by generic names and the mean number of drugs per prescription.

In this regard, several interventions have been taken by the Ministry of Health of Uganda through establishment of a list of essential medicines, revitalisation of Medicines and Therapeutics committees at hospitals (Ministry of Health Uganda, 2018), issuance of clinical guidelines (Ministry of Health Uganda, 2016) and regularly reviewing the performance of the pharmaceutical sector (Ministry of Health Uganda, 2015).

The Medicine and Therapeutics Committees (MTC) have been trained and provided with MTC manuals to guide them on how to assess the prescription patterns within the hospital. However, the extent to which prescribers adhere to the treatment guidelines and the influence they might have on pharmacy services to the patients is not yet assessed in many hospital settings of Uganda. Therefore, the purpose of this study was to assess the effects of prescription patterns on the performance of pharmacy department of Mbarara Regional Referral Hospital, Uganda.

## Methods

### Study design and setting

The study was a mixed method and of analytical cross-sectional design, conducted in January and February 2023 at the outpatient department (OPD) and OPD pharmacy of Mbarara Regional Referral Hospital (MRRH). MRRH is located in South-western Uganda in Mbarara Municipality along Mbarara-Kabale road. It is a tertiary level of care with a staffing of 386 health workers. It offers a wide range of specialised curative and preventive services ranging from psychiatric care to ENT, radiology, pathology, ophthalmology, obstetrics and gynaecology. It caters mostly for the Western Region districts of Mbarara, Bushenyi, Ntungamo, Kiruhura, Ibanda and Isingiro (A Brief History of Mbarara Regional Referral Hospital, 2023). The OPD was selected for the study because that is where the real situation about stock outs and various prescription malpractices can be more evident compared to inpatient department.

### Study population and eligibility criteria

The OPD of MRRH which was the place of study was composed of 86 health workers, of whom 3 were clinical officers, 3 Dentists, 11 Medical Officers, 29 Medical Interns, 13 midwives and nurses, 15 senior house officers and 12 specialists. All the health workers in the OPD were eligible for the study because they are variably engaged in prescribing medicines. Furthermore, the OPD of MRRH, for a period of 5 years (2018–2022) received an average of 2401 out patients per month, therefore these were taken as the target OPD population. To be included in the study, the eligible health workers had to provide consent to participate. Outpatients included in the study had to possess a prescription from the MRRH OPD, consented to participate and being able to actually sign the consent form. Inpatients on treatment or those discharged from MRRH were excluded from the study.

### Sample size and sampling procedures

The number of health workers in the OPD to be included in the study was determined by the census method whereby data was collected from all the 86 legally recognised prescribers at the OPD. In this study, given that there was no previous work on the number of OPD patients who leave the hospital with no drugs, Yamane formula was used to estimate the sample size based on the average of 2401 OPD patients received at MRRH per month (Kasiuleviius et al., 2006). The sample size of patients was rounded off to 300 for logistical reasons.

### Data collection instruments, procedures and quality control

The instruments used in this study were adopted from the WHO standardised tools for drug use indicators and the medicines and the Therapeutics Manual of 2018 by the Uganda Ministry of Health (Ministry of Health Uganda, 2018; WHO, 1993). Likewise, extensive literature review of similar studies was carried out to identify some variables used to enrich the instruments. The majority of the responses in this study tools were in binary mode of Yes or No.

The prescribers’ questionnaire probed into the designation, level of qualification, experience, time spent at facility, accessibility and reference to guidelines namely Essential Medicines List (EML) and Uganda Clinical Guidelines (UCG) during prescription of medicines to patients. The patients’ questionnaire was structured in two sections. Section 1 provided details of patient’s demographics including age, gender and residential status in relation to the catchment area. Section 2 captured a wide range of information from the prescription including source of prescription, cadre of prescriber, number of medicines prescribed, and prescription by either generic or brand name, prescription by EML, stock out and the prescription fill rate.

Data collection was done using the questionnaires. Research assistants were trained to fill the questionnaire. To collect data from the patients, the research assistants were deployed at the various OPD dispensing points. Data were collected from the patients after obtaining informed consent from them and ethical clearance reference number MUST/2022-685 was granted by Mbarara University of science and technology . Section 2 of the patients’ questionnaire was completed with information extracted from the prescription of the patient. For prescribers (i.e. clinicians), data were obtained from their respective workstations in the absence of patients to avoid interruption during work and to ensure confidentiality. All the activities were performed under the supervision of the principal investigator.

Pretesting of the instruments was done for 3 days at the OPD before the beginning of the study. It involved 15 patients not be included in the main study, who came with prescriptions to collect medicines from the OPD pharmacy and consented to participate. To ensure internal consistency, ambiguous sections were corrected afterwards in accordance with the feedback received for improved clarity. The same was done for the prescribers’ instrument, whereby 10 prescribers not forming part of the OPD team were asked to complete the tool, after which corrections were made accordingly.

### Data processing, study variables and analysis

Data were recorded in excel sheet and exported for analysis into IBM SPSS Statistics for Windows version 26.0 (IBM Corp, Armonk, NY, USA) for analysis. Descriptive statistics were generated, summarised as proportions and means, and presented in frequency tables and graphs. To identify the variables that influence prescription fill rate (the dependent variable), bivariate and multivariable logistic regression analyses were done. Factors with *p*-values less than 0.05 in bivariate analysis were further subjected to multivariable logistic regression analysis. A *p*-value of less than 0.05 was considered statistically significant. Odds ratios with 95% confidence intervals were determined to show the strength of the associations.

## Results

### Prescribers in the outpatient department

The results from the responses of the prescribers in the OPD are as presented in Table 1.

**Table 1.** Characteristics of the prescribers (*N* = 86).

Variables	Frequency *n*	Percentage %
Designation of prescriber		
Clinical officers	3	3.5
Dentists	3	3.5
Medical officers	11	12.8
Medical interns	29	33.7
Midwives/nurse	13	15.1
Senior House officer	15	17.4
Specialists	12	14.0
Highest level of qualification		
Bachelors	54	62.8
Diploma	13	15.1
Masters	17	19.8
Others	2	2.4
Current clinic deployment		
General OPD	25	29.1
Paediatric	12	14.0
Special clinics	26	30.2
MCH	8	9.3
NCD clinics	4	4.7
Emergency	11	12.8
Years of experience		
0–5 years	29	33.7
6–15 years	15	17.4
>15 years	42	48.8
Years worked at the facility		
0–5 years	34	39.5
6–15 years	14	16.3
>15 years	38	44.2
Awareness of the existence of UCG or EML (*n* = 86)		
No	10	11.6
Yes	76	88.4
Access to UCG or EML if you need (*n* = 76)		
No	17	22.4
Yes	59	77.6
Refer to UCG or EML while making prescription (*n* = 59)		
No	7	11.9
Yes	52	88.1
Frequency of referring to UCG or EML while making prescription (*n* = 52)		
Case by case	11	21.2
Every prescription	2	3.8
Once in a while	39	75.0
Attended a CPD on appropriate medicine use in the last 6 months		
No	53	61.6
Yes	33	38.4
Get updates on stock status from the pharmacies stores		
No	25	29.1
Yes	61	70.9
Frequency of receipt to stock status updates (*n* = 62)		
Every fortnight	4	6.2
Every month	22	35.5
Every week	31	50.0
Every day	5	8.1
Reference to the updates		
No	24	27.9
Yes	62	72.1

The medical interns were the most prescribers at 33.7% (19), followed by Senior House Officers at 17.4% (15); and the least were the Dentists and Clinical Officers at 3.5% (3) each. The distribution of prescribers by qualification shows that 62.8% (54) had bachelor’s degree, 19.8% (17) masters, 15.1% (13) diploma and other qualifications only 2.4% (2). With regard to prescribers’ current clinic of deployment, majority 30.2% (26) and 29.1% (25) were deployed in the specialised clinics, and in general OPD respectively. Most prescribers (48.8%, 42) had a work experience of more than 15 years, followed by 33.7% (29) who had the shortest work experience of up to 5 years. Majority (44.2%, 38) had worked at the health facility for more than 15 years, followed by 39.5% (34) who had worked for up to 5 years. Results on awareness of the existence of the UCG or EML show that most (88.4%, 76) were aware of the existence of UCG or EML, but the rest 11.6% (10) had no idea of their existence. Furthermore the results show that there was variation in accessing UCG or EML for those who were aware of them, whereby 77.6% (59/76) had UCG or EML, while 22.4% (17/76) did not. Concerning making reference to the UCG or EML (for those who had them) while preparing prescriptions, 88.1% (52/59) did so but 11.9% (7/59) never referred to them. About the frequency of referring to UCG or EML (for those who referred to them) while prescribing, 75.0% (39/52) did so once in a while, followed by 21.2% (11/52) who referred to them case by case, and 3.8% who did it for every prescription. With respect to attending CPD on appropriate medicine use in the last 6 months, it was found that 61.6% had not attended CPD but 38.4% had attended. The distribution of frequency of receipt of weekly stock status updates from pharmacy stores was as follows: 70.9% received updates but 29.1% did not. Out of those who got updates from pharmacy stores 50% reported receiving it weekly. As for those who received stock status updates 72.1% referred to them while 27.9% did not.

### Patient demographics and content of their prescriptions

About 64.0% (192/300) prescriptions came from patients aged below 50 years and 108 (36%) from patients aged 50 years and above. Prescriptions from patients residing within the MRRH catchment area comprised 97.7% (293/300) and the rest came from outside the area.

Most of the prescriptions received at various data collection points came from medical officers at 69.7% (209/300), followed by dental officer at 18.3% (55/300), and clinical officers at 5.7% (17/300), with the rest of prescribers including specialists contributing considerably much less proportions.

**Figure 1.**
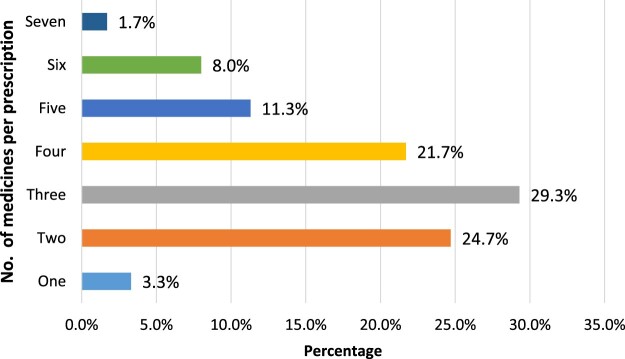
Number and percentage of medicines per prescription.

As for the number of medicines, 29.3% (88/300) prescriptions had three medicines, 24.7% (74/300) two medicines, 21.7% (65/300) four medicines, 11.3% (34/300) five medicines, 8.0% had six medicines, 3.3% had one medicine and 1.7% had seven medicines. The mean number of medicines per prescription was 3.437 (SD = 1.361) (Figure 1).

**Figure 2.**
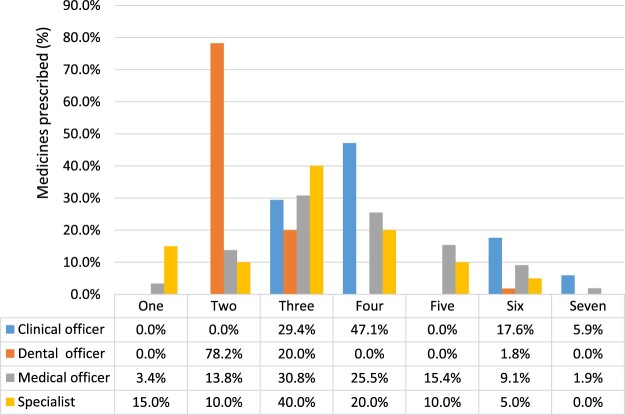
Number of medicines prescribed by each clinical staff cadre.

The number of medicines prescribed by the different clinical staff cadres was heterogeneous but showed a pattern of being higher for the two to four medicine prescriptions (Figure 2). Most notable is the fact that majority (15%) of single medicine prescriptions were made by the specialists, two medicines (78%) by the dental officers, three medicines (40%) again by specialists, four medicines (47.1%) by the clinical officers, five (15.4%), six (17.6%) and 7 (5.9%) respectively by the medical officers and clinical officers (Figure 2).

#### Adherence to essential medicines list

Out of the 300 prescriptions reviewed, 76% adhered to the EML guideline while 24% did not. Clinical-cadre-specific EML adherence shows that 94.5% of the dental officers adhered to EML, followed by 80% of specialists, 71.6% of medical officers and 64.7% of clinical officers. It was noted that 62% adhered to WHO recommendation of prescribing by generic name as opposed to 38% who did not adhered. About 87.3% of the prescriptions made by dental officers had generic names, as were 70% of those made by specialists, 55.3% by medical officers and 52.9% by clinical officers.

### Prescription fill rates (dependent variable)

Results generated from the prescriptions showed that the prescription fill rate was 60.5%. This was further disaggregated into 68.6% of the prescriptions being partially dispensed, 29.7% not filled and only 1.7% fully filled. The analysis further went on to establish how many drugs were not issued and it was seen that 32.7% of the prescribed medicines were fully dispensed, 22.3% of the prescription which had one medicines not dispensed, 22.7% were two, 14% had three medicines, 6% had four medicines, 2.3% had 5 medicines from the prescription not dispensed.

Prescription fill rate was high among dental officer (COR: 4.846, 95% CI: 1.630-14.408, *p *= 0.005) as compared to specialists, while low prescription fill rate was registered by clinical officers (COR: 0.094, 95% CI: .010–0.854, *p *= 0.036) and medical officers (COR: 0.368, 95% CI: 0.141–0.959, *p *= 0.041) compared to specialists. Furthermore, low prescription fill rate was registered among those who did not use generic names (COR: 0.337, 95% CI: 0.191–0.593, *P* < 0.001) and those who did not adhere to EML (COR: 0.370, 95% CI: 0.188–0.728, *p *= 0.004) as shown in Tables 2 and 3.

**Table 2.** Bivariate analysis of characteristics to determine their influence on prescription fill rate (i.e. proportion availed).

Variables	All prescriptions availed		COR (95% CI)	*p-*value
	Yes (%)	No (%)		
Cadre of the prescribers				
Clinical officers	16 (7.7%)	1 (1.1%)	0.094 (0.010–0.854)	0.036
Dental officers	13 (6.2%)	42 (45.7%)	4.846 (1.630–14.408)	0.005
Medical officers	167 (80.3%)	41(44.6%)	0.368 (0.141–0.959)	0.041
Specialists	12 (5.8%)	8 (8.7%)	1.0	
Total	208 (69.3%)	92 (30.7%)		
Prescribing by generic names				
No	94 (45.2%)	20 (21.7%)	0.337 (0.191–0.593)	<0.001
Yes	11 (54.8%)	72 (78.3%)	1.0	
Adherence to EML				
No	60(28.8%)	12 (13.0%)	0.370 (0.188–0.728)	0.004
Yes	148(71.2%)	80 (87.0%)	1.0

**Table 3.** Multivariable analysis of characteristics to determine their influence on prescription fill rate.

Variables	COR (95% CI)	*p*-value	AOR (95% CI)	*p*-value
Cadre of the prescribers				
Clinical officers	0.094 (0.010–0.854)	0.036	0.105 (0.011–0.970)	0.047
Dental officers	4.846 (1.630–14.408)	0.005	4.303 (1.426–12.989)	
Medical officers	0.368 (0.141-0.959)	0.041		0.010
Specialists	1.0		0.403 (0.152–1.064)1.0	0.067
Prescribing by generic names				
Yes	0.337 (0.191–0.593)	<0.001	0.569 (0.298–1.083)	0.086
No			1.0	
Adherence to EML				
Yes	0.370 (0.188–0.728)	0.004	0.714 (0.336–1.520)	0.383
No	1.0		1.0	

When the factors were analysed in multivariable model to control for cofounding and interactions, the cadre of the prescribers was found to be significantly associated with prescription fill rates. Being dental officer (COR: 4.303, 95% CI: 1.426–12.989, *p *= 0.010) was associated with higher prescription fill rate compared to specialists. However, being clinical officer (COR: 0.105, 95% CI: 0.011–0.970, *P* = 0.047) was associated with low prescription fill rate.

## Discussion

Prescription pattern is a factor that affects the performance of pharmacies in several key dimensions relating to medicine availability and patients’ satisfaction. These were assessed in this study based on a sample of 300 patient encounters that took place at the OPD in Mbarara RRH.

The study findings indicate that the average number of medications ordered per prescription was 3.437. This is higher than the WHO standard (1.6–1.8) (Desalegn, 2013; Jain et al., 2015). The deviation from WHO standard has been reported by similar studies that were conducted in developing countries such as those carried out in Sudan, Zimbabwe and Nigeria that reported the average number of medications ordered per prescription to be 1.4, 1.3 and 3.8 respectively (Bilal et al., 2016). These differences in average medicines per prescription could be attributed to various factors such as nature of cadres at OPD, and commitment to adherence to reference standards.

It is important to recall that only 76% of the prescribers adhered to the EML guideline which is lower than the 100% adherence to EML recommendation by WHO and the Uganda treatment guideline (FMHACA, 2014). The non-adherence to treatment guidelines recorded in this study is higher than one reported in Zambian hospitals which showed adherence to EML of 57.65% (Adisa et al., 2016), but still lower than that of the Colombo group of tertiary care teaching hospitals [(National Hospital of Sri Lanka (NHSL), Lady Ridgeway Hospital (LRH) for Children, and De Soysa Hospital for Women (DSHW)] in which between March and August 2015 adherence was at 91.1% (Galappatthy et al., 2021). Low adherence could be due to several factors such as lack of availability of guidelines as it was reported by 30.2% of prescribers in this study; and lack of awareness as was found in this study whereby some prescribers had not heard of the existence of EML and UCG.

The ineffective communication between prescribers and pharmacy department could be another factor that could have contributed to non-adherence to guidelines since some of the prescribers were not aware of the updated list of pharmaceutical products available in the pharmacy stock. Furthermore, 69.8% had access to UCG and EML and 30.2% couldn’t access them. In addition most of cadres (61.6%) did not participate in CPD on appropriate medicine use while CPDs help the healthcare professional to remain updated, catalyse the career development and the safeguard of the patient.

It is important to note that the notion of EML utilisation is hinged on the fact that the utilisation of a limited number of well-researched and cost-effective medicines will result in better health care, increased long-term medicines supply plus more equitable and sustainable access to health products. The non-adherence to the guidelines and prescription errors has been associated with different factors such as inadequate knowledge or competence, an unsafe working environment, complex or undefined procedures, and inadequate communication among health-care personnel, particularly between doctors and nurses.

Different cadres prescribed a varying number of medicines (Figure 2). According to the figure, 15% of the prescription with one medicine were prescribed by specialists, 78% of the prescription of two medicines were prescribed by dental officers, 47.1% of the prescriptions with four medicines were prescribed by clinical officers, 15.4% of the prescriptions with five medicines were done by medical officers and 17.5% of the prescription with six medicines were done by clinical officers. While in this research 33.7% of prescribers had 0–5 years of experience, previous studies have shown that the medical training and prescribers’ experience were associated with prescribing patterns.

WHO strongly recommends generic prescription since it helps to enhance communication and clarity amongst healthcare workers and also aids in the reduction of treatment costs for the patient (Desalegn, 2013; Ofori-Asenso, 2016). In this study, the percentage of medicines prescribed with generic name is 62% (Galappatthy et al., 2021), which is lower than the rate identified in Sri Lanka and South East Asia (90.1%), though all are lower than the WHO 100% recommendation. The comparison of different cadres in relation to the index of rational drug prescription shows that 87.3% of the prescription done by dental officers had generic names, 70% of the prescription done by specialists had generic names, 55.3% of the prescription done by medical officer had generic names and 52.9% of the prescription done by clinical officers had generic names.

In this study, the prescription fill rate was 60.5% which was lower than 100% recommended by WHO. However, the type of profession (cadre) grossly affected the prescription fill. Being dental officer (COR: 4.303, 95% CI: 1.426–12.989, *p *= 0.01) was significantly associated with good prescription fill compared to specialist. However, being clinical officer (COR: 0.105, 95% CI: 0.011–0.970, *p *= 0.047) was highly associated with low prescription fill rate. This may be explained by the observation that most of the clinical officers were stationed in Out Patient Department where medicine promoters visit most frequently which could affect their prescription pattern; and possibly not due to their level of training. This is consistent with study (Hangoma, 2014) which found that the association between prescribers’ level of training and the factors that influence prescribing tendencies and fill rate were not statistically significant (*p *= 0.084) (28). The low fill rate could be associated with factors such as funding gap, poor quality of prescriptions and drug stock outs.

## Conclusion

The overall prescription fill rate was 60.5%, being higher among dental officers and lower among those who did not adhere to generic prescribing and EML. Most of the prescribers had prescribed three medicines per prescription, 62% of the prescription used generic names and 76% of the prescriptions adhered to EML. About 68.7% of the prescriptions were partially dispensed. The study findings indicate that the cadre of the prescribers was significantly associated with prescription fill rate, higher among dental officers and low among clinical officers.

In light of the above, analysis of prescription patterns, the non-full compliance to the good prescriptions patterns affected the performance of the pharmacy department of an MRRH. This was evident in the low prescription fill rate while, ideally, all patients who come to MRRH pharmacy should leave with all their respective prescriptions filled.

Thus these findings highlight that the Medicines and Therapeutics Committee of the hospital could prioritise and implement the quality improvement strategies to address deviations from the national guidelines and the WHO standards. There is need to increase awareness about the EML, UCG and availing them to enhance their access by the clinical prescribers. Sustained monitoring of the prescription patterns should be strengthened to ensure that the national and international prescription conventions/guidelines are upheld.

## List of abbreviations

RRH – Regional Referral Hospital

MRRH – Mbarara Regional Referral Hospital

PP – Prescription Patterns

OPD – Out Patient Department

UCG – Uganda Clinical guidelines

EML – Essential Medicines List

EMHS – essential Medicines and Health Supplies

WHO – World Health Organisation

EAC RCE–VIHSCM: EAC Regional Centre of Excellence for Vaccines, Immunisation, and Health Supply Chain Management

## Declarations

### Ethics approval and consent to participate

Ethical clearance for this study was sought from and granted by MUST and it has reference number MUST/2022-685 of 3rd February 2023. All participants of the study were informed about the objectives and anonymity of the study and informed consent was granted by the participants before the start of the study. All the information collected was kept confidential and only used for the purpose of the study.

### Consent for publication

Not applicable.

### Availability of data and materials

The datasets used during the current study are available from the corresponding author on reasonable request.

## APPENDIX 4: DATA COLLECTION TOOL 1 Questionnaires on why prescribers prescribe drugs outside the Essential Drug list.

DATA COLLECTION TOOL 2: patient’s demography.
